# Bacteriophages as antimicrobial agents against major pathogens in swine: a review

**DOI:** 10.1186/s40104-015-0039-7

**Published:** 2015-08-25

**Authors:** Jiancheng Zhang, Zhen Li, Zhenhui Cao, Lili Wang, Xiaoyu Li, Shuying Li, Yongping Xu

**Affiliations:** School of Life Science and Biotechnology, Dalian University of Technology, Dalian, 116024 China; Ministry of Education Center for Food Safety of Animal Origin, Dalian, 116600 China; Faculty of Animal Science and Technology, Yunnan Agricultural University, Kunming, 650201 China; Dalian SEM Bio-Engineering Technology Co. Ltd., Dalian, 116620 China

**Keywords:** Antibiotic resistance, Bacteriophage, Food safety, Phage therapy, Swine

## Abstract

In recent years, the development of antibiotic resistant bacteria has become a global concern which has prompted research into the development of alternative disease control strategies for the swine industry. Bacteriophages (viruses that infect bacteria) offer the prospect of a sustainable alternative approach against bacterial pathogens with the flexibility of being applied therapeutically or for biological control purposes. This paper reviews the use of phages as an antimicrobial strategy for controlling critical pathogens including *Salmonella* and *Escherichia coli* with an emphasis on the application of phages for improving performance and nutrient digestibility in swine operations as well as in controlling zoonotic human diseases by reducing the bacterial load spread from pork products to humans through the meat.

## Background

In the past two decades, bacterial diseases caused by pathogens such as *Escherichia coli* and *Salmonella* have become a major issue for the swine industry [1]. Antibiotics commonly used worldwide represent a relatively efficient way to eliminate infectious pathogens [2]. However, recent studies indicate that the abuse of antibiotics has led to several negative effects such as antibiotic residues in meat products and the development of antibiotic resistant bacteria [3, 4].

With many countries banning the use of antibiotics to control bacterial infections in swine, studies on alternatives with antimicrobial effects have become increasingly popular [3, 4]. Among the various alternatives available (i.e. probiotics, prebiotics, oligosaccharides, antimicrobial peptides and essential oils), phages are starting to receive increased attention due to their special characteristics, such as widespread distribution, self-replication and a lack of effects on the normal microflora of treated animals [5]. In this paper, we review the results and findings of recent studies regarding the application of phages in swine production including a discussion of their benefits and their potential use as a pre/post-slaughter disease control strategy.

## What are bacteriophages?

Bacteriophages are viruses that affect bacteria. Phages are very common in all natural environments and play an important role in bacterial evolution [6]. Virulent phages can be isolated from sources such as swine feces, waste water and soil indicating that they are fairly widespread in commercial swine facilities and therefore, it should be easy to obtain phages specific for many of the diseases present in most swine operations [7]. In our previous study, Niu et al. [8] recovered phages in 239 of 855 samples (26.5 % of 411 pooled fecal pats, 23.8 % of 320 fecal grab samples, 21.8 % of 87 water trough samples, and 94.6 % of 37 pen floor slurry samples). Studies in feedlot calves indicate that environmental factors such as moisture level and temperature influenced the presence of *E. coli* O157:H7 phage [9].

Phages can be categorized into two types, namely virulent (exclusively undergo the lytic cycle) and temperate (are able to endure the lysogenic cycle) [10]. Phages are very specific as each type generally attacks different bacterial species. Virulent phages enter the bacterial cell, replicate using the host machinery and finally lyse the host cell, leading to the disintegration of the bacteria. In contrast, temperate phages enter the cell and instead of creating new phage particles, the phage DNA first integrates into the bacterial chromosome to produce a prophage. The formed prophage replicates each time the host cell divides. Eventually a stimulus such as ionizing radiation or a specific chemical induces the prophage to initiate the lytic cycle. Temperate phages can’t be used as antimicrobial agents for therapeutic purposes, as they may transfer genetic material from one bacterial cell to another. This may result in unpredictable horizontal gene transference such as toxic or antibiotic resistance genes which may cause detrimental effects on therapy. In contrast, virulent phages rapidly exterminate the bacteria, enabling them to be used as efficient antibacterial agents [11].

## Use of phages in the swine industry

Although phage therapy has been used successfully in swine since the early 1920’s [12], it has only recently started to attract the attention of the research community as a tool for use against bacterial diseases in swine. These endeavors have resulted in a renewed interest in phages as a means of preventing and treating bacterial diseases in swine operations [4]. The objectives of using phage therapy in the swine industry include reducing the impact of infectious diseases caused by several bacterial pathogens on animal health and production as well as controlling zoonotic human pathogens by reducing the bacterial load spread from swine to humans through pork [13]. Moreover, there is increased interest in the use of phages for post-harvest control of bacterial microorganisms in both pork products and processed foods [14].

## Use of phages to improve pig performance and nutrient digestibility

Three investigations about the effects of phage therapy on pig performance are summarized in Table 1. Yan et al. [15] reported that dietary supplementation with anti-*Salmonella* phage had no effect on average daily gain (ADG) or gain:feed (G:F) of growing pigs. However, Kim et al. [16] reported an improved ADG and average daily feed intake (ADFI) with increasing dietary phage supplementation but there was no effect on G:F. Gebru et al. [17] observed an improvement in ADG and a decrease in G:F in *Salmonella* challenged pigs fed diets supplemented with 3 × 10^9^ PFU/kg anti-*Salmonella* typhimurium phage. The difference in results is believed to be associated with differences in the level and type of phage investigated, health status within herds, farm hygiene, diet composition, feed form and interactions with other dietary feed additives.

**Table 1:** Effects of dietary supplementation with phages on pig performance

Diets		ADG, g	ADFI, g	G:F	Reference	Aims
BD^1^		459	1284	0.36	Yan et al. [15]	Evaluate the effects of *Salmonella* phages on the performance of growing pigs
BD + 22 ppm tylosin		464	1231	0.38		
BD + 0.025 % phage		455	1294	0.35		
BD + 0.05 % phage		472	1272	0.37		
BD		737	2079	0.35	Kim et al. [16]	Effects of dietary supplementation with phages, probiotics and a combination of the two on pig performance
BD + 0.5 g/kg phage		764	2129	0.36		
BD + 1.0 g/kg phage		815	2240	0.36		
BD + 1.5 g/kg phage		822	2222	0.37		
Before challenge^4^	CON^2^	654	1688	0.39	Gebru et al. [17]	Effects of dietary supplementation with probiotic, anti- *Salmonell*a typhimurium phage, organic acid combinations, or fermented soybean on pig performance
	AST^3^	627	1652	0.38		
After challenge	CON	273^a^	1,313^a^	0.21^a^		
	AST	719^b^	1,938^b^	0.08^b^		

^1^BD = Basal diet. ^2^CON = control diet with no added antimicrobial

^3^AST = 3 × 10^9^ PFU/kg anti-*Salmonella* typhimurium phage supplementation

^4^Values are calculated for the 2 weeks before *Salmonella* typhimurium challenge and the 2 weeks after challenge

^a,b^Means in the same column with different superscripts differ (*P* < 0.05)

Results obtained in nutrient digestibility experiments indicate that pigs fed diets supplemented with phage have greater nutrient digestibility (Table 2). Yan et al. [15] reported an improved dry matter, nitrogen and energy digestibility for growing pigs fed diets supplemented with 0.025 and 0.05 % anti-*Salmonella* phage. In agreement with Yan’s work, Kim et al. [16] reported a small increase in dry matter and energy digestibility with increasing concentrations of phage from 0.5 to 1.5 g/kg.

**Table 2:** Effects of phages on nutrient digestibility in pigs

Diets	Dry matter	Nitrogen	Energy	Crude protein	References
BD^1^	0.774^b^	0.770^b^	0.766^b^		Yan et al. [15]
BD + 22 ppm tylosin	0.801^a^	0.784^ab^	0.792^a^		
BD + 0.025 % phage	0.793^a^	0.801^a^	0.778^ab^		
BD + 0.05 % phage	0.796^a^	0.792^a^	0.785^ab^		
BD	0.841		0.874	0.831	Kim et al.[16]
BD + 0.5 g/kg phage	0.846		0.875	0832	
BD + 1.0 g/kg phage	0.847		0.875	0.838	
BD + 1.5 g/kg phage	0.852		0.879	0.845	

^1^BD = Basal diet

^a,b^Means in the same column with different superscripts differ (*P* < 0.05)

It is well known that the microflora in the gastrointestinal tract play a number of important roles in swine production because the intestine is an important nutrient absorption site. Therefore, a possible reason for the increased digestibility observed in phage treated pigs is likely to be their improved bacterial profile in the gut. Reduced populations of *Salmonella* and coliform and increased numbers of *Lactobacillus* and *Bifidobacterium,* which have been observed in fecal microflora investigations in pigs fed diets supplemented with phage [16], are in agreement with the results of pigs fed diets supplemented with anti-*Salmonella* phage [17].

Table 3 shows the anti-*Salmonella* and anti-coliform activity resulting from increased levels of phage supplementation. The increased numbers of *Lactobacillus* and *Bifidobacterium* are believed to make a large contribution to the improvement in pig performance and nutrient digestibility observed in other experiments. Wall et al. [18] reported that administration of anti-*Salmonella* phage to pigs challenged with *Salmonella* could reduce *Salmonella* colonization in the ileum and cecum by 90 to 99.9 %.

**Table 3:** Effects of phages on fecal microflora numbers (log_10_ CFU/g) in pigs

Diets	Lactobacillus	Bifidobacterium	Coliforms	Salmonella	References
BD^1^	6.89^b^		6.55^a^	3.62^a^	Yan et al. [15]
BD + 22 ppm tylosin	6.93^b^		6.00^b^	2.57^b^	
BD + 0.025 % phage	7.16^ab^		6.32^ab^	2.21^b^	
BD + 0.05 % phage	7.52^a^		6.14^b^	2.02^a^	
BD	8.56	8.92	8.57		Kim et al. [16]
BD + 0.5 g/kg phage	8.67	9.37	8.22		
BD + 1.0 g/kg phage	9.06	9.77	7.77		
BD + 1.5 g/kg phage	8.98	9.75	7.84		

^1^BD = Basal diet

^a,b^Means in the same column with different superscripts differ (*P* < 0.05)

## Therapeutic uses of phages

### *Salmonella* infections

*Salmonella*, which is responsible for severe diarrhea in humans, is considered as one of the most common food and water-borne pathogens in the world and a variety of serum types have been separated from the different stages of the pig production process [19]. It is a fact that the increasing incidence of *Salmonella* being isolated from healthy finishing swine threatens food safety and limits meat export opportunities from pork-producing countries [20].

Human *Salmonellosis* is typically associated with cross-contamination and temperature/time abuse of meat products in which *Salmonella* can reach numbers sufficient to cause infections in the human body [21]. Owing to increasing reports of antimicrobial resistance in *Salmonella,* the importance of controlling this pathogen by finding alternatives to the use of antibiotics to reduce *Salmonella* in swine should be stressed [20].

Different types of *Salmonella* phages have been isolated from effluent lagoons, sewage and feces of swine [22]. A high abundance of *Salmonella* phage was observed in swine effluent lagoons, including one study which reported levels as high as 2.1 × 10^9^ PFU/mL [22]. In addition, phages active against *Salmonella* typhimurium were isolated from 1 % of the individual fecal samples which showed that phage populations might vary in accordance with *Salmonella* populations [19].

To date, at least 25 *Salmonella* phage genomes have been reported, in which the genome size ranged from 33 to 240 kb [14]. This indicates that a variety of *Salmonella* phages exist in nature. The presence and diversity of phages in a variety of environments indicates that specific phages with high virulence could be easily obtained which may help the development of *Salmonella* pathogen reduction strategies in the swine industry.

Several virulent phages have been used to reduce the concentration of various species of *Salmonella*, including Enteritidis and Typhimurium [23, 24]. Most recently, a phage cocktail was used to reduce the *S. ty*phimuriumγ4232 in artificially-infected market-weight swine and *S. t*yphimuriumγ4232 was reduced by 2–3 log_10_ CFU [25]. Several reports suggest that treatment with a large number of phages is desirable and there was no evidence to suggest that the highest possible concentrations of phages should not be used [26].

Albino et al. [27] isolated a *Salmonella* phage belonging to the *Podoviridae* family, which significantly reduced (*P* < 0.05) *Salmonella* at a relatively low concentration (10^7^ PFU/mL) in an *in vitro* experiment. However, the *in vivo* results were not statistically significant in any of the analyzed intestinal locations (ileum, cecum, feces), although *Salmonella* was detected in the feces of challenged animals after treatment with phages at a concentration of 10^7^ PFU/mL. The reason for the low activity of phage *in vivo* may be due to an inappropriate micro-ecology in the animal’s gut. In another study, a significant reduction of *Salmonella* typhimurium concentration in several tissues was observed by Lee and Harries [28] in an experiment in which piglets were fed a single broad-spectrum virulent phage.

### *E. coli* O157:H7 infections

Since *E. coli* O157:H7 was identified in 1983 [29], it has been recognized as an important zoonotic human pathogen. Previous outbreaks of *E. coli* O157:H7 infection resulted from food, water and direct fecal contact [30]. Infection with *E. coli* O157:H7 resulted in diarrhea, hemorrhageic colitis, hemolytic-uremic syndrome and thrombotic thrombocytopenic purpura [31]. Moreover, *E. coli* O157:H7 have been associated with numerous diseases such as bloody diarrhea and hemolytic uremic syndrome in humans [32]. Previous studies on phages, primarily used to control pathogenic *E. coli* in pigs, calves and lambs [33] achieved very promising results.

A relatively abundant *E. coli* O157:H7 phage was isolated from swine feces in a recent study [34]. Morita et al. [32] investigated a swine stool sample which contained 4.2 × 10^7^ PFU/g of the *E. coli* O157:H7 specific phage PP01, indicating that phage PP01 might suppress its host *E. coli* O157:H7 in the gastrointestinal ecosystem.

Several studies have evaluated the antimicrobial ability of phages targeted against *E. coli.* Smith and Huggins [33] investigated the efficacy of a two-phage mixture against infection induced by the ETEC strain P433 in neonatal pigs. In an *in vitro* experiment, both phages showed a high capacity to lyse bacteria with nine particles of P433/1 and four particles of P433/2 required to completely lyse broth cultures of their respective hosts. In addition, the results of this work indicated that phages that targeted colonizing pili (F4, F5, F6 or F18) were more effective in controlling a larger proportion of the porcine ETEC than phages that target other pili [33].

A study using anti-ETEC phage therapy in swine was conducted by Jamalludeen et al. [11]. Six phages lysing the ETEC strain O149:H10:F4 and three phages lysing the ETEC strain O149:H43:F4 were isolated with 10 strains of ETEC used in total. For 85 strains of O149:H10 ETEC, Phage GJ1-GJ6 lysed 99–100 % of them, while for 42 strains of O149:H43 ETEC, only 0–12 % strains were lysed by phage GJ1-GJ6. Three other phages (GJ7-GJ9) selected against an O149:H43 host strain lysed 86–98 % of 42 strains of O149:H43 and 2–53 % of strains of O149:H10 [11]. Subsequently, phages GJ1-GJ7 were individually evaluated for their ability to treat an experimental infection with an O149:H10:F4 enterotoxigenic *E. coli* in weaned pigs. A significant reduction in the severity of diarrhea and the composite diarrhea score was observed in a prophylactic treatment supplemented with a combination of three phages, which indicates that the selected phage cocktail was effective in controlling the experimental ETEC strain O149:H10:F4 [11].

Similar to the application of phage in pigs, Waddell et al. [35] showed successful elimination of *E. coli* O157:H7 in experimentally inoculated (10^9^ CFU) calves through the oral administration of 10^11^ PFU of a mixture of six phages on days −7, −6, −1, 0 and 1 post-inoculation with pathogenic *E. coli* O157:H7. The results obtained with pigs and calves reinforce the idea that treatments with multiple doses and different administration times are important in effective phage therapy, which will make significant differences to the effectiveness of phages.

## Use of phages to increase food safety

One important source of food contamination by *E. coli* O157:H7 is the transmission of the bacterium from feces onto meat during slaughter [36]. O’Flynn et al. [37] evaluated whether a phage cocktail could be used to remove or decrease bacteria on meat carcasses. A phage cocktail which consisted of phages e11/2, e4/1c, and pp01 was pipetted medially onto nine slices of meat contaminated with a rifampin-resistant derivative of *E. coli* O157:H7 strain P1432. Among those samples that were treated with phage cocktails, seven of the nine samples were completely free of *E. coli* O157:H7, which was determined by a viable plate count after enrichment. However, control pieces of meat were positive, exhibiting counts of *E. coli* O157:H7 of 10^5^ CFU/mL [37]. Although this research was conducted with cattle, it indicates that the surface application of phages is a feasible approach for food preservation and could also be applied to pork.

A phage cocktail (PC1), able to lyse a variety of *S. enterica,* was modified to use the broad host-range phage Felix O1 and three phages isolated from sewage. The cocktail of PC1, which was applied to pig skin artificially-contaminated with multi-drug resistant *S.* typhimurium U288, produced a significant (*P* < 0.05) decrease in *S.* typhimurium U288 (Table 4) [14]. The use of a MOI in excess of the bacterial concentration seems to be closely related to the effectiveness of the treatment. Bacterial counts were at undetectable levels after the application of PC1 to pig skin (>99 % reduction). In this research, the low temperature (4 °C) required for meat storage did not decrease the passive action of the phage. This result indicates that the contaminating *Salmonella* could be eliminated by phage before potential exposure of consumers to meat products.

**Table 4:** Mean log_10_ CFU counts of *Salmonella* typhimurium U288 recovered from experimentally-contaminated 4 cm^2^ pig skin sections of control and bacteriophage cocktail PC1 treated samples

U288 inoculum, CFU	Phage inoculum, PFU			Untreated controls
Sample time	10^7^	10^5^	10^4^	
*1 h*				
10^6^	6.2 ± 0.1	6.1 ± 0.2	6.2 ± 0.2	6.2 ± 0.1
10^4^	3.5 ± 0.1*	3.7 ± 0.2*	4.6 ± 0.1	4.7 ± 0.2
10^3^	3.8 ± 0.1	3.3 ± 0.4	3.4 ± 0.1*	4.2 ± 0.2
*48 h*				
10^6^	5.0 ± 0.1*	5.9 ± 0.2	6.5 ± 0.2	6.3 ± 0.1
10^4^	2.9 ± 0.4*	3.9 ± 0.1*	4.1 ± 0.1	4.3 ± 0.1
10^3^	3.6 ± 0.2	3.6 ± 0.4	ND	4.1 ± 0.2
*96 h*				
10^6^	*5.5* ± 0.2*	6.6 ± 0.2	6.7 ± 0.1	6.5 ± 0.2
10^4^	3.2 ± 0.3*	3.4 ± 0.2*	4.1 ± 0.4	4.5 ± 0.1
10^3^	2.8 ± 0.7	ND	ND	4.3 ± 0.3

Hooton et al. [14]. ND = not detectable; *P < 0.01 compared with control values

## Problems associated with the use of phages

Although phage therapy has many advantages, previous research suggests that the use of phages exhibit some disadvantages [11, 38]. Firstly, phages have a narrow range of hosts resulting in a limitation of their use for broad-spectrum protection [11]. In addition, it is possible to have an immune response to the administered phages in the animal body [39]. Finally, bacteria resistance to the virulent phage can be caused by phage and bacteria co-evolution [39]. However, due to rapid developments in the field of phage therapy, it is hoped that all limitations which currently exist will soon be resolved.

According to the results shown in previous work, phages are unstable in the stomach and upper small intestine. The results [9, 33] obtained with the application of orally administered phages in infected animals including piglets suggest phages are sensitive when exposed to a low pH (~pH 2), but showed considerable stability at a high pH. Additional research should focus on the need for protective strategies within the gastrointestinal tract for the administration of phages, such as microencapsulation to allow the phages to adapt to a wider pH range [40, 41].

Ma et al. [40] evaluated the development of a microencapsulated phage Felix O1 for oral delivery using a novel chitosan-alginate-CaCl_2_ system. In this study, the viability of free and encapsulated phages when they were subjected to simulated gastric fluid and bile salts was compared. A large proportion of phage Felix O1 micropheres retained their biological activity in a simulated gastrointestinal tract environment which indicates that the encapsulation technique may help the phages survive at a low pH in the stomach and then subsequently act in the small intestine. In addition, Brussow [42] suggested that administration of phages immediately after feeding was a promising strategy in order to avoid exposure to a low pH in the stomach. However, a low pH will not cause a serious problem in young animals because they have a higher pH in their stomach [26].

Phage sensitivity to temperature is another important factor which could affect the effectiveness of phages in animals. A previous study [43] reported that the *in vitro* virulence of most phages tested declined drastically at 24 °C, and sometimes at 20 °C, suggesting that environmental temperature could be a limiting factor in determining the ability of phages to multiply outside the animal body. In fact, Smith et al. [43] reported that the virulence of some temperature sensitive phages was reduced around 37 °C, which is the normal body temperature for most animal species including pigs. They suggested that selection of phage mutants that were not so sensitive to temperature could be of value in overcoming the negative effects of temperature on the effectiveness of phage therapy. Moreover, utilization of microencapsulated phages showed an optimistic result to prevent the degradation of phage particles from high temperatures in a previous *in vivo* study [9, 33].

Another factor that may attenuate phage activity is the rapid development of phage-resistance. Phage resistance might result in three ways including the blocking of phage receptors, the production of an extracellular matrix and the production of competitive inhibitors. In addition, other mechanisms such as preventing phage DNA entry, cutting phage nucleic acids and abortive infection systems play an important role in phage-resistance as well [6]. Compared with the antibiotic-resistance developed by pathogens, phage therapy seems to face similar problems as the rapid development of phage resistance could reduce their infective efficiency. However, diversity of phage types and the likelihood of quick phage isolation increases the feasibility of new phage cocktail development. The use of cocktails consisting of various phages with the use of different bacterial receptors has been proposed to circumvent resistance problems [44].

## Conclusions

Phage therapy shows significant potential to be used as a viable strategy to restrain and cure infectious diseases caused by major pathogens in the swine industry. A number of commercially produced phage products have been approved to be used as bio-control agents in the field of poultry raising, cattle breeding, and food preservation [45–48]. However, the use of phages is still limited in controlling food borne pathogens in live animals as well as in understanding the mechanism through which they improve pig performance. Without an understanding of the essential problems including phage resistance, phage-host interactions, the microbial ecosystem, and the host animal, this biological pathogen control system will not be used to its fullest potential in improving swine production.
